# Sexual Offending: Cognition, Emotion, and Motivation

**DOI:** 10.5964/ejop.v14i1.1525

**Published:** 2018-03-12

**Authors:** David Prescott

**Affiliations:** a LICSW, Clinical Services Development Director, Becket Family of Services, Orford, NH, USA

[Other p1]Many will remember, and many are still living with it: In the 1980s, professionals often assumed that repeat sex offenders followed a “cycle” into re-offense. In an interview with the New York Times, Barbara Schwartz displayed remarkable candor in acknowledging how many of our field’s original approaches were created in response to momentary needs and not based in science. The various proposed cycles, often available in materials designed for a program and then shared with colleagues via workbooks, chapters, and conference presentations, all centered on the idea that a person would experience some life event, often the result of seemingly unimportant decisions, and that they would develop ideas and fantasies about some sort of illegal sexual behavior. This may seem over-simplified, but it is nonetheless the take-home lesson of many professionals at the time (in the author’s experience). Although this cycle originally started – and was intended – as a helpful guide for understanding the process by which sex crimes occurred, it became a reified construct in many treatment programs, despite there being no research to support its use in that context. Many professionals approached this cycle in the way originally intended; many more did not.

Unintended consequences of this single-cycle approach included the belief that all sexual offenders would follow the same pathway to re-offense and that to some extent all were specialists in how they abused. For many, a byproduct of assuming that a “cycle” existed was the belief that re-offense was inevitable in most cases without intervention. Of course, many of these unintended consequences were not the fault of the cycle so much as the result of professionals not yet having a rich body of research from which to devise treatment approaches.

In the late 1990s, Tony Ward came onto the scene, observing (along with his colleague, the late Stephen Hudson) that some people who sexually abused are motivated to do so, while others are not. Some people, they found, are very explicit in planning their offenses, while other crimes are more opportunistic. People following each of these pathways to offending typically believe their actions are justified and often recall their crimes as positive experiences. However, others do not intend to sexually abuse anybody, but may find themselves in situations where they lack adequate skills to prevent their crimes. This model of the offense process, known as the self-regulation model, has received considerable empirical study (e.g., Kingston, Yates, & Firestone, 2012; Kingston, Yates, & Olver, 2014).

Understanding that the roles of cognition and motivation can vary dramatically from one person who sexually abuses to the next, Ward went on to develop the Good Lives Model as a rehabilitative framework for people who have abused. In one of the most influential articles on this model, Ward teamed up with the lead editor of the current volume, Theresa Gannon (Ward & Gannon, 2006). They would go on to write a scathing, yet influential, article on the current state of treatment within the criminal justice system, titled *“Where has all the psychology gone?”* (Gannon & Ward, 2014). Along the way, Ward established himself as a brilliant commentator on the theories underlying the assessment and treatment of people who sexually abuse. An implicit message of his work is that professionals must move beyond measures and protocols to a deeper understanding of each client and an empirically informed means of doing so. Gannon, meanwhile, has researched fascinating areas on the periphery of our field, from topics related to deception to fire setting. Together, they have brought out the best in one another in their published works.

This latest volume sees Gannon and Ward assemble a diverse panel of experts to explore the interaction of cognition, emotion, and motivation in sexual offending. Returning the focus to emotional elements after decades of scrutinizing cognitive and behavioral elements is certainly welcome. Ward starts the volume with a chapter on the enactment of emotions. He describes the connection between emotions and brain functions, with their subsequent effect on thoughts and behavior. He places this into the context of states (situations in which emotions occur) and traits (e.g., personality disorders), then explores how emotional states are enabled by events. He next describes “affective framing”, the process by which people interpret the events, situations, themselves, and others as “embodied desiderative feelings”. In other words, the enactment of emotional states is a whole-body experience related to a wish to achieve a particular state of being.

Why is this important? Clearly, Ward’s contribution matters because most treatment programs in the criminal justice world employ simplified cognitive-behavioral models, methods, and techniques (some would argue overly simplified). Although research offers a strong basis for these approaches, many at the front lines of treatment have felt that this is not enough in every case. It is one thing to discuss the thoughts related to emotions, and it is another to address their primacy in the lives of people who have abused. Further, in this author’s professional experience, people who have abused very often do everything they can to avoid experiencing their emotions. Indeed, it can often seem that they lack the curiosity to observe their experience in general (Levenson, Willis, & Prescott, 2017).

In some cases, this estrangement from emotions can appear as a kind of script: “The last time I approached something that I truly desired (a child), I ended up hurting it as well as a lot of other people, including myself. Therefore, I don’t want to feel anything like desire in my life.” In other cases, their own experience of abuse may have left them with the belief that experiencing desire and other emotions could be toxic. Often, these people spent so much of their lives scanning their environment for threats that they became unable to experience their feelings and emotions. If programs are to prevent re-offending, directly addressing emotional enactment may be vital to client well-being as well and, by extension, to public safety (Levenson et al., 2017).

From this point, the book turns to many authors who explore related themes. Sarah Brown contributes a chapter on bridging the divide between emotions and cognitions by exploring empathy and its place in treatment. She emphasizes the shortcomings to date of interventions that attempt to enhance empathy among people who have abused. Jill Stinson’s chapter looks into motivation and self-regulation, noting the strength of each in the etiology of offending. Geris Serran provides a chapter exploring historical approaches to treatment and offering ideas for integrating knowledge of emotions, cognitions, and behavior into a more seamless approach. Numerous other chapters provide unique perspectives, all in the direction of making treatment more meaningful and effective for people who have sexually abused others.

As always, Theresa Gannon and Tony Ward find themselves at the leading edge of theory and practice in the assessment and treatment of those who sexually abuse. Not surprisingly, the book is written in dense prose such that readers may need to work to grasp and fully appreciate the concepts presented (one ATSA member once described Ward’s work as “chewy”). The authors make no apologies for this, and given the objectives of this volume there is no reason they should. This book is for people who want to understand the forefront of theory and practice.
